# The Growth and Ion Absorption of Sesbania (*Sesbania cannabina)* and Hairy Vetch (*Vicia villosa*) in Saline Soil Under Improvement Measures

**DOI:** 10.3390/plants13233413

**Published:** 2024-12-05

**Authors:** You Wu, Rui Liu, Wei Si, Jiale Zhang, Jianhua Yang, Zhenxin Qiu, Renlei Luo, Yu Wang

**Affiliations:** 1 College of Energy and Power Engineering, Lanzhou University of Technology, Lanzhou 730050, China; liurui9982023@163.com (R.L.); siwei197@163.com (W.S.); zhjiale97@163.com (J.Z.); yangjianhua82@163.com (J.Y.); qiuzhenxin0405@163.com (Z.Q.); luorenlei1230@163.com (R.L.); 2 Ministry of Agriculture and Rural Affairs Smart Agriculture Irrigation Equipment Key Laboratory, Lanzhou 730050, China; 3 Lanzhou Institute of Husbandry and Pharmaceutical Sciences of Chinese Academy of Agricultural Sciences, Lanzhou 730050, China; wangyu02@caas.cn

**Keywords:** saline-alkali soil, ion absorption, sesbania, hairy vetch, sheep manure, gravel, straw

## Abstract

Soil salinization is a serious threat to the ecological environment and sustainable agricultural development in the arid regions of northwest China. Optimal soil salinization amelioration methods were eagerly explored under different soil salinity levels. Sesbania and hairy vetch are salt-tolerant plants, and green manure improved the saline environment. In this study, two leguminous halophytic crops, sesbania (*Sesbania cannabina*) and hairy vetch (*Vicia villosa*), were planted under different salinity levels, combined with three saline soil improvement measures, namely gravel mulching, manure application, and straw returning. No improvement measures and no salinity treatment was set as the control (CK). This study was conducted to analyze the effects of soil salinization improvement measures on the growth and ion uptake of sesbania and hairy vetch as biological measures under different soil salinity levels. Sesbania under manure application absorbed the highest soil Na^+^ (2.71 g kg^−1^) and Cl^−^ (2.66 g kg^−1^) amounts at a soil salinity of 3.2 g kg^−1^, which was 14.7% and 10.95% higher than under gravel mulching and straw returning, respectively. Na^+^ and Cl^−^ absorption of hairy vetch under manure application reached the highest value of 1.39 g kg^−1^ and 1.38 g kg^−1^ at a soil salinity of 1.6 g kg^−1^, which was 24.46% and 22.31% higher than under gravel mulching and straw returning, respectively. Plant height and stem diameter as well as root growth and development of sesbania and hairy vetch were limited at soil salinities greater than 1.6 g kg^−1^ and 0.8 g kg^−1^. Overall, sesbania and hairy vetch effectively absorbed both soil Na^+^ and Cl^−^ under manure application, thus regulating soil salinity and reducing soil salinization. However, soil salinity levels greater than 3.2 g kg^−1^ and 1.6 g kg^−1^ not only weakened the ionic absorption capacity but also inhibited the growth and development of sesbania and hairy vetch. This study provides evidence that soil salt ion absorption by sesbania and hairy vetch is promoted effectively, ameliorating soil salinity, under manure application as compared to under gravel mulching and straw returning.

## 1. Introduction

Soil salinization is a major factor limiting agricultural production and sustainable agricultural development in arid and semi-arid zones and poses a serious threat to the ecological environment [1,2,3,4]. It is noteworthy that there is already more than 1.1 × 10^9^ hm^2^ of salinized land in the world, and this figure is increasing year by year. China’s existing saline land area is approximately 3.6 × 10^7^ hm^2^, being one of the countries with the widest distribution of saline land in the world; Northwest China is the region with the most extensive distribution of saline land [5,6,7]. Therefore, soil salinization alleviation has become a goal that has been pursued with urgency.

Generally, there are three main methods to alleviate salinized soil, including engineering and chemical and biological improvement measures [8]. However, engineering improvement measures utilizing the principle of water–salt movement are not sustainable in terms of resource savings, especially in water-scarce areas. In addition, gravel mulching reduces salinity aggregation in soil surfaces, inhibits soil secondary salinization, and is an effective way to prevent and control soil salinization. The results of Liu et al. [9] showed that gravel mulching effectively reduces soil salinity. Chen et al. [10] found that the desalination rate of straw returning to the field reached 53%; the salt ions Na^+^ and Cl^−^ were reduced by 52.7% and 51.9%, respectively. Moreover, manure application improves saline soil mainly by affecting the movement of soluble salts and water-soluble ions in the soil, which effectively reduces the salt concentration of saline surface soils [11]. Wang et al. [12] showed that the application of organic fertilizer was beneficial to slow down the rate of soil salinization. Furthermore, the effects of halophytic crops on saline soil environments mainly occur through the physiological function of halophytic crops and root infiltration in salt absorption and salt secretion, as a way to reduce soil salinity [13]. Although the current research shows that all of the above improvement measures can effectively alleviate soil salinization, exploring the potential of different soil salinization improvement measures under various soil salinity levels is still urgently needed.

Halophytic crops have gradually surfaced as one of the most effective, fundamental, and long-term saline soil improvement measures. Among them, sesbania and hairy vetch have strong salt-tolerant abilities and are excellent plants for alleviating saline in soils. Sarwar et al. [14] proposed that planting sesbania significantly reduced the salinity of salinized soil, and the root secretion of sesbania increased the soil Ca^2+^ content, inhibited the accumulation of Na^+^, and promoted the formation of benign soil structure. After planting sesbania in saline soil, the total salt content of the soil decreased by 1.30 g kg^−1^ [15]. In addition, through the uptake characteristics of salt-tolerant plants in rhizosphere soil, it was found that sesbania had the most obvious effect on the removal of Na^+^ in saline soil [16]. Additionally, hairy vetch not only improves land and water utilization but also alleviates saline soil [17]. Previous results showed that soil salinity reduced by 71.1% in the 100 cm soil layer [18]. In recent years, research on sesbania and hairy vetch has focused on molecular, rhizobacterial, breeding, and genetic aspects [19,20,21], as well as the effect of sesbania hairy vetch as a green manure on the yield of the subsequent crop and the effect of leguminous green manure on soil fertility [22,23]. However, little is still known about the effects of sesbania and hairy vetch on the desalination of saline soils under different soil salinity levels.

Amending soil salinization is a major challenge. For this reason, this study set up six soil salinities levels and three amelioration methods (manure application, gravel mulching, and straw returning) to study soil salt ion changes as well as the growth and ion uptake of sesbania and hairy vetch. The objectives of this study were to further explore the soil salinization alleviation potential of sesbania and hairy vetch as biological measures coupled with other soil salinization improvement measures (manure application, gravel mulching, and straw returning) under different soil salinity levels, to propose the appropriate soil salinity ranges for optimal soil salinization improvement measures, and finally to provide theoretical support for soil salinization alleviation with various soil salinity levels. We hypothesized that (1) the growth and development of sesbania and hairy vetch would be beneficial under low salinity conditions and (2) improvement measures would be more favorable for the absorption of soil ions by sesbania and hairy vetch.

## 2. Results

### 2.1. Growth Indexes of Sesbania and Hairy Vetch

#### 2.1.1. Plant Height and Stem Diameter

**Figure 1:**
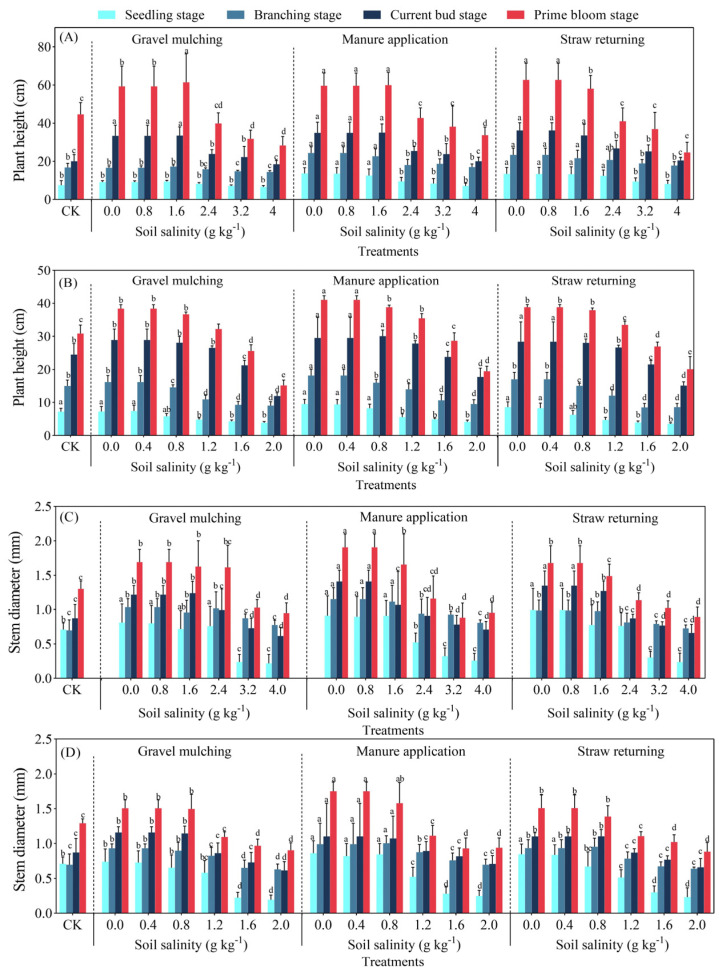
Plant height and stem diameter of sesbania and hairy vetch under different soil salinity improvement measures. (**A**,**C**) Sesbania; (**B**,**D**) hairy vetch. CK, soil salinity of 0.0 g kg^−1^ without any soil salinity improvement measures. Different lowercase letters indicate that there were significant differences among the 19 treatments in the same growth stage at the *p* < 0.05 level.

With the increase of soil salinity levels, the plant height of both sesbania and hairy vetch gradually decreased (Figure 1). At a soil salinity of 0.8 g kg^−1^, the highest plant height at seedling, branching, current bud, and prime bloom stage of sesbania under manure application was 11.28, 23.22, 35.35, and 58.67 cm, which increased by 17.9%, 18.7%, 19.26%, and 19.56% compared with gravel mulching, and 10.11%, 14.45%, 18.72%, and 19.01% compared with straw returning, respectively. The plant height of hairy vetch was the highest in all the four growing periods under manure application at a soil salinity of 0.4 g kg^−1^, which was 10.73, 19.95, 31.36, and 41.41 cm, respectively. The plant height of both sesbania and hairy vetch showed manure application > straw returning > gravel mulching > control treatment (CK). Manure addition had the most significant effect on the promotion of plant height in sesbania and hairy vetch. 

The stem diameter of both sesbania and hairy vetch decreased gradually with increasing soil salinity levels (Figure 1). The stem diameters of sesbania at seedling, branching, current bud, and prime bloom stages were 0.83, 1.27, 1.56, and 1.85 cm under manure application, respectively. The stem diameters of hairy vetch at seedling, branching, bud-bearing and current bud stages were 0.69, 1.02, 1.44, and 1.86 cm under manure application, respectively. Compared with CK, at a soil salinity greater than 0.4 g kg^−1^ and 0.8 g kg^−1^, the stem diameter of sesbania and hairy vetch, respectively, was significantly inhibited (*p* < 0.05). The combined analysis showed that the stem diameter of sesbania and hairy vetch was higher under manure application than under gravel mulching and straw returning.

#### 2.1.2. Dry Matter Mass and Root–Shoot Ratio

**Figure 2:**
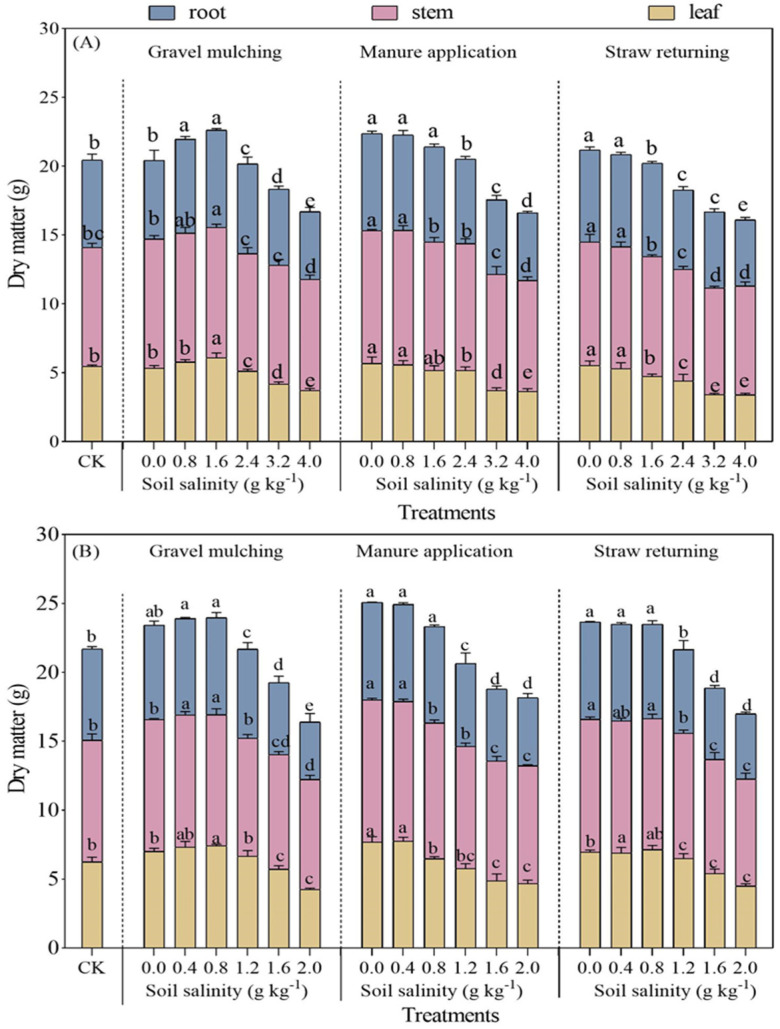
Dry matter mass of sesbania and hairy vetch under different salinity and saline improvement measures. (**A**) Sesbania; (**B**) hairy vetch. CK, soil salinity of 0.0 g kg^−1^ without any soil salinity improvement measures. Different lowercase letters indicate that there were significant differences among the 19 treatments in the same organ of sesbania or hairy vetch at the *p* < 0.05 level.

The dry matter mass of both sesbania and hairy vetch decreased with increasing soil salinity levels (Figure 2). Compared with CK, the aboveground and belowground dry matter mass of sesbania under M increased by 12.69% and 23.20%, respectively. Similarly, at a soil salinity level of 0.4 g kg^−1^, the aboveground and underground dry matter mass of hairy vetch under manure application respectively increased by 13.24% and 11.88% compared with CK. However, both sesbania and hairy vetch dry matter mass decreased sharply with increasing soil salinity levels. At a soil salinity level of 4.0 g kg^−1^, the aboveground dry matter mass of sesbania was 9.42, 10.81, and 9.87 g under gravel mulching, manure application, and straw returning, which decreased by 30.27%, 21.95%, and 27.32% compared with CK, respectively. At soil salinity level of 2.0 g kg^−1^, the aboveground dry matter mass of hairy vetch significantly decreased, by 30.15% 25.15%, and 30.14% under gravel mulching, manure application, and straw returning, compared with under CK, respectively (*p* < 0.05). Manure application promoted the biomass accumulation of sesbania and hairy vetch to some extent.

**Figure 3:**
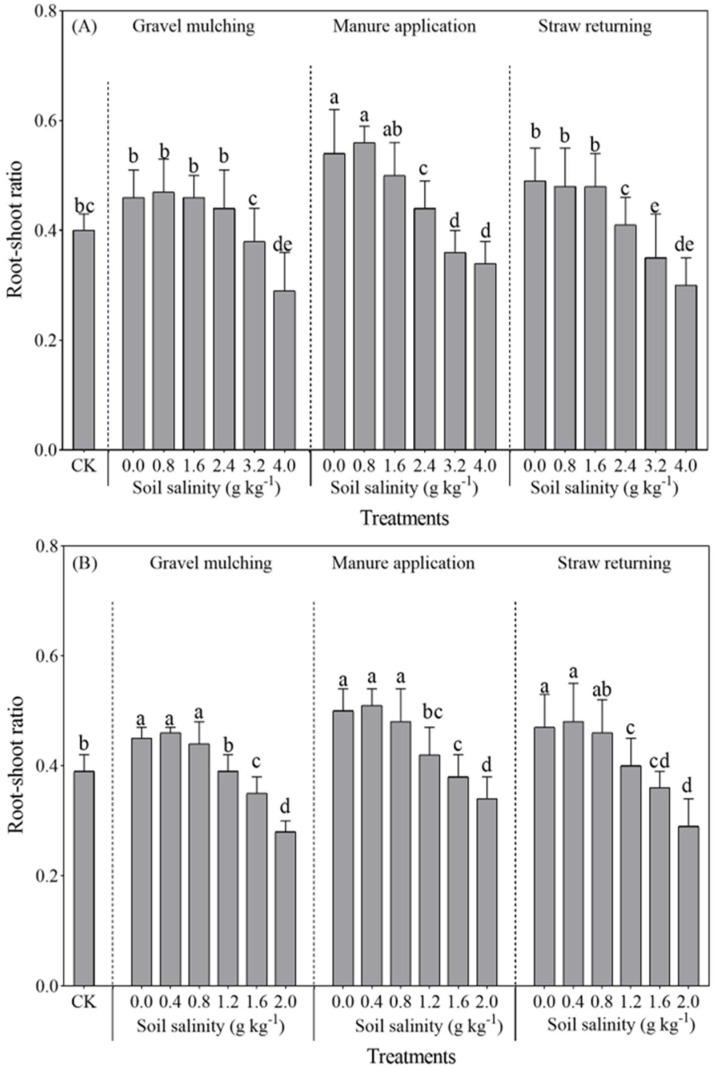
Root–shoot ratio of sesbania and hairy vetch under different salinity and saline improvement measures. (**A**) sesbania; (**B**) hairy vetch. CK, soil salinity of 0.0 g kg^−1^ without any soil salinity improvement measures. Different lowercase letters indicated that there were significant differences among the 19 treatments at the *p* < 0.05 level.

Compared with under CK, the root–shoot ratio of sesbania was not significantly different under SC0.8 and SC1.6 (Figure 3). However, the root–shoot ratio differed significantly under manure application and straw returning, compared to under CK (*p* < 0.05). At a soil salinity level of 4.0 g kg^−1^, the root–shoot ratios of sesbania were only 0.34, 0.38, and 0.34 under gravel mulching, manure application, and straw returning, which decreased by 30.61%, 20.83%, and 25.53% compared with under CK, respectively. At a soil salinity level of 2.0 g kg^−1^, the root–shoot ratios of hairy vetch under gravel mulching, manure application, and straw returning were 0.38, 0.39, and 0.38 which, compared with under CK, decreased by 26.92%, 17.02%, and 19.15%, respectively. Soil salinity above 0.8 g kg^−1^ severely inhibited the roots and aboveground growth of hairy vetch, leading to significant changes in the root–shoot ratio.

#### 2.1.3. Leaf Area

**Figure 4:**
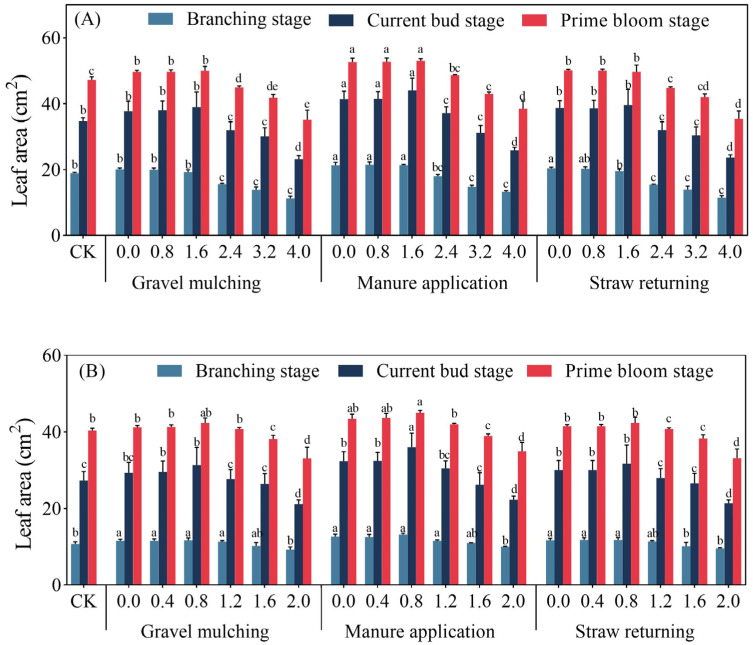
Leaf area of sesbania and hairy vetch under different salinity and saline soil improvement measures. (**A**) Sesbania; (**B**) hairy vetch. CK, soil salinity of 0.0 g kg^−1^ without any soil salinity improvement measures. Different lowercase letters indicate that there were significant differences among the 19 treatments in the same growth period at the *p* < 0.05 level.

At soil salinity levels of 0.8 and 1.6 g kg^−1^, there was no significant difference in the leaf area of sesbania at the branching stage between gravel mulching, manure application, and straw returning (Figure 4). The leaf area of sesbania at the present current bud stage had no significant difference between gravel mulching and straw returning (Figure 4A). At a soil salinity of 1.6 g kg^−1^, the leaf area of sesbania reached a maximum value of 53.2 cm^2^ under manure application at the prime bloom stage, which was 7.89% and 7.52% higher than under gravel mulching and straw returning. At the branching stage of hairy vetch, there was no significant difference in the leaf area between gravel mulching and straw returning at the same soil salinity (*p* > 0.05). The highest leaf area of hairy vetch at the present current bud stage was obtained at a soil salinity of 0.8 g kg^−1^ under gravel mulching, manure application, and straw returning, which was 30.1, 36.1, and 31.8 cm^2^, respectively. Under manure application, the leaf area of hairy vetch under 0.4 g kg^−1^ soil salinity was 43.7 cm^2^, which increased 15.38% and 13% as compared with gravel mulching and straw returning, respectively. In addition, at soil salinities of 1.6 and 2.0 g kg^−1^, the leaf area of hairy vetch in prime bloom was not significantly different between gravel mulching, manure application, and straw returning. Overall, at soil salinity levels of 1.6 g kg^−1^ and 0.8 g kg^−1^, the leaf area indices of sesbania and hairy vetch under different treatments (gravel mulching, manure application, and straw returning) were higher than those of other salinity levels. Manure application significantly increased the leaf area indices of sesbania and hairy vetch at soil salinity levels of less than 1.6 g kg^−1^ and 1.2 g kg^−1^, respectively.

### 2.2. Root Growth of Sesbania and Hairy Vetch

#### 2.2.1. Root Lengths

**Figure 5:**
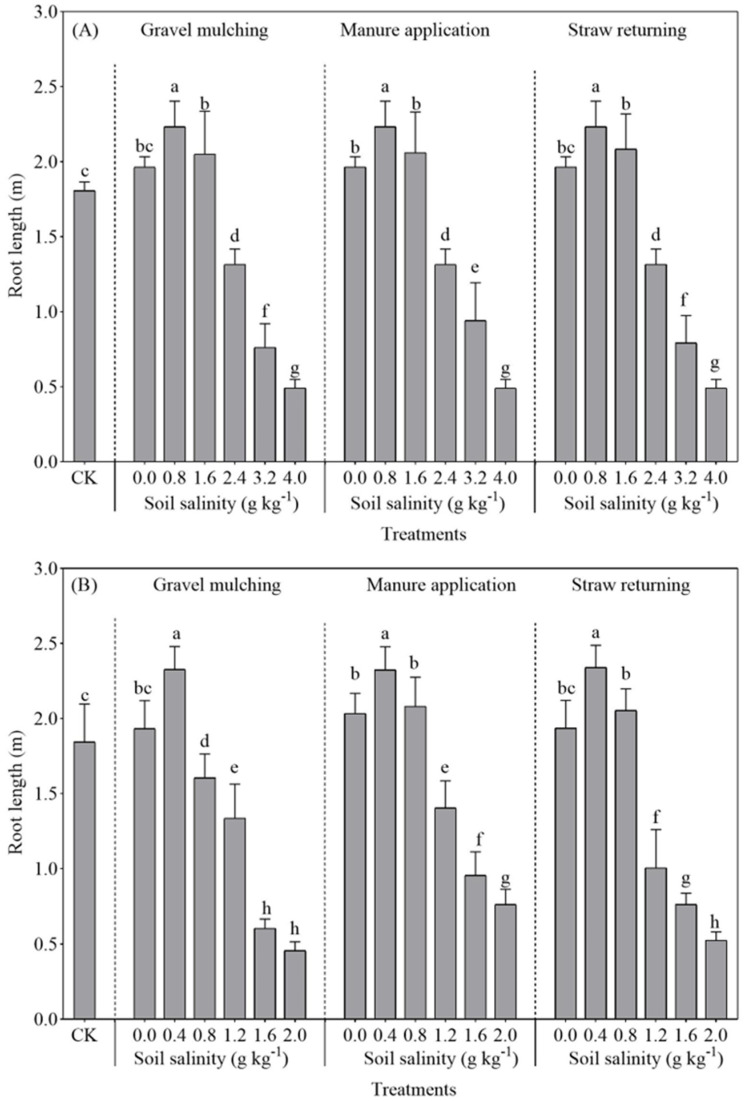
Root length of sesbania and hairy vetch under different salinity and saline improvement measures. (**A**) Sesbania; (**B**) hairy vetch. CK, soil salinity of 0.0 g kg^−1^ without any soil salinity improvement measures. Different lowercase letters indicate that there were significant differences among the 19 treatments at the *p* < 0.05 level.

With the increase of soil salinity, the root length of both sesbania and hairy vetch decreased significantly (Figure 5A,B). At a soil salinity level of 0.8 g kg^−1^, the root lengths of sesbania under gravel mulching, manure application, and straw returning were 2.33 m, 2.32 m, and 2.34 m, respectively. When soil salinity increased to 1.6 g kg^−1^, the root length of sesbania under gravel mulching was 1.61 m, which was significantly decreased by 22.87% and 28.76% compared with manure application and straw returning (Figure 5A; *p* < 0.05). With increasing soil salinity, the hairy vetch root length under manure application gradually decreased and reached a maximum value of 2.51 m at a soil salinity level of 0.4 g kg^−1^, increasing by 16.93% and 15.73% compared with gravel mulching and straw returning (Figure 5B).

#### 2.2.2. Root Diameter

**Figure 6:**
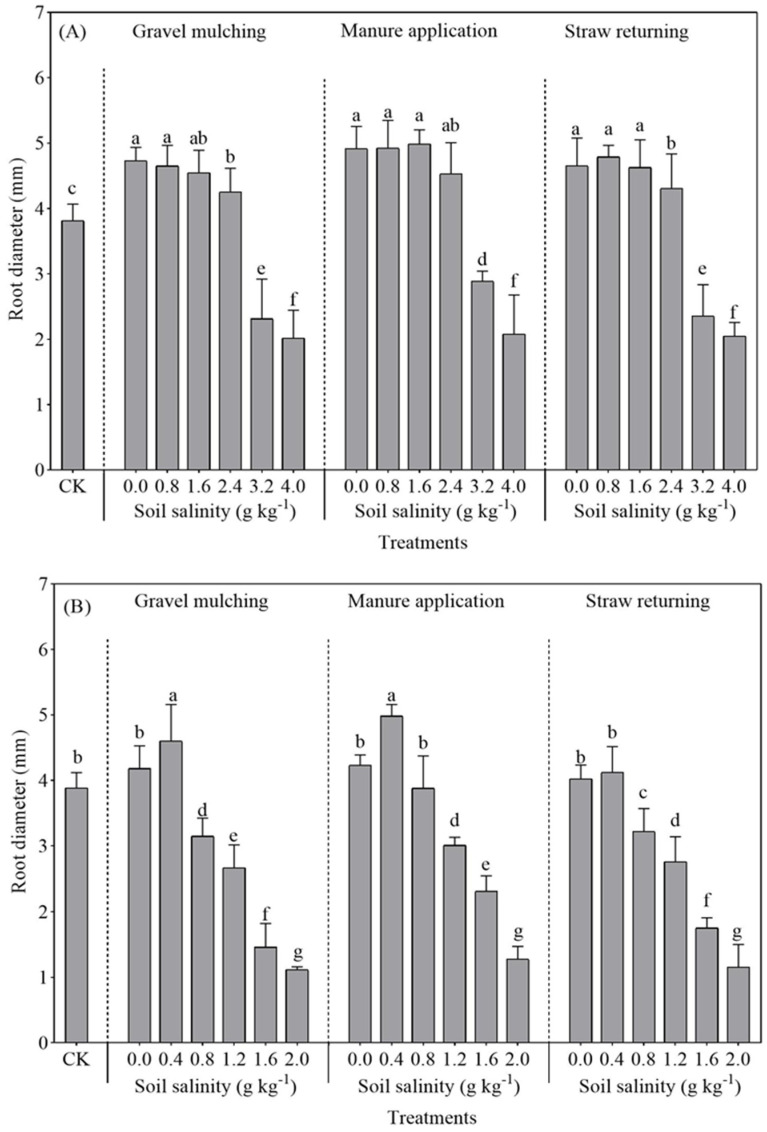
Root diameter of sesbania and hairy vetch under different salinity and saline improvement measures. (**A**) Sesbania; (**B**) hairy vetch. CK, soil salinity of 0.0 g kg^−1^ without any soil salinity improvement measures. Different lowercase letters indicate that there were significant differences among the 19 treatments at the *p* < 0.05 level.

At a soil salinity of 0.0–0.8 g kg^−1^, the root diameter of sesbania reached the maximum value under gravel mulching and manure application: 4.55 and 5.09 mm (Figure 6A). Overall, the maximum root diameter of hairy vetch under gravel mulch, manure application, and straw return was found at a soil salinity of 0.4 g kg^−1^. Among them, the application of manure made the hairy vetch root diameter reach 4.986 mm, representing an increase of 11.83% and 13.19% compared to gravel mulching and straw returning, respectively. A soil salinity greater than 0.4 g kg^−1^ significantly decreased the root diameter of hairy vetch. There were no significant changes in the sesbania root diameter at soil salinities of 0.0–1.6 g kg^−1^. When soil salinity exceeded 1.6 g kg^−1^, the sesbania root diameter gradually decreased.

#### 2.2.3. Root Volume

**Figure 7:**
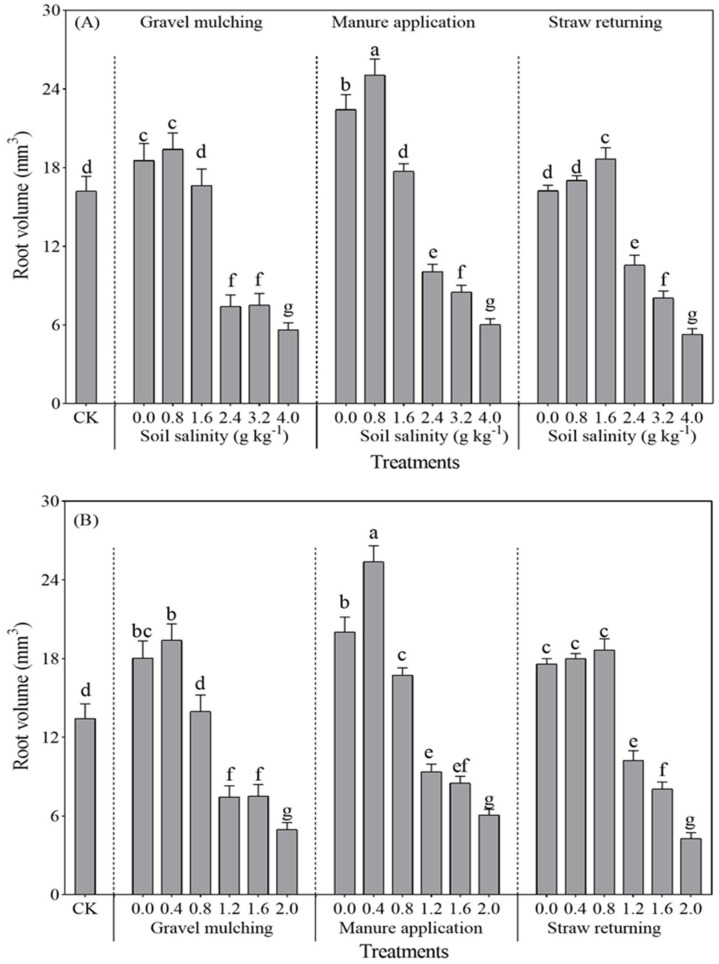
Root volume of sesbania and hairy vetch under different salinity and saline amelioration measures. (**A**) Sesbania; (**B**) hairy vetch. CK, soil salinity of 0.0 g kg^−1^ without any soil salinity improvement measures. Different lowercase letters indicate that there were significant differences among the 19 treatments at the *p* < 0.05 level.

With the increase of soil salinity, the root volume of both sesbania and hairy vetch gradually decreased (Figure 7). Compared with CK, the sesbania root volume at a soil salinity of 0.8 g kg^−1^ increased by 10.64%, 26.33%, and 14.54% under gravel mulching, manure application, and straw returning (Figure 7A). The root volume and root surface area of hairy vetch were similar to those of sesbania. The root volume of hairy vetch peaked at a soil salinity of 0.4 g kg^−1^, with the greatest increase in root volume under manure application. At a soil salinity of 0.4 g kg^−1^, manure application increased the root surface area of hairy vetch by 10.93% and 11.44%, and the root volume by 23.56% and 20.72%, compared with gravel mulching and straw returning, respectively (Figure 7B).

#### 2.2.4. Root Surface Area

**Figure 8:**
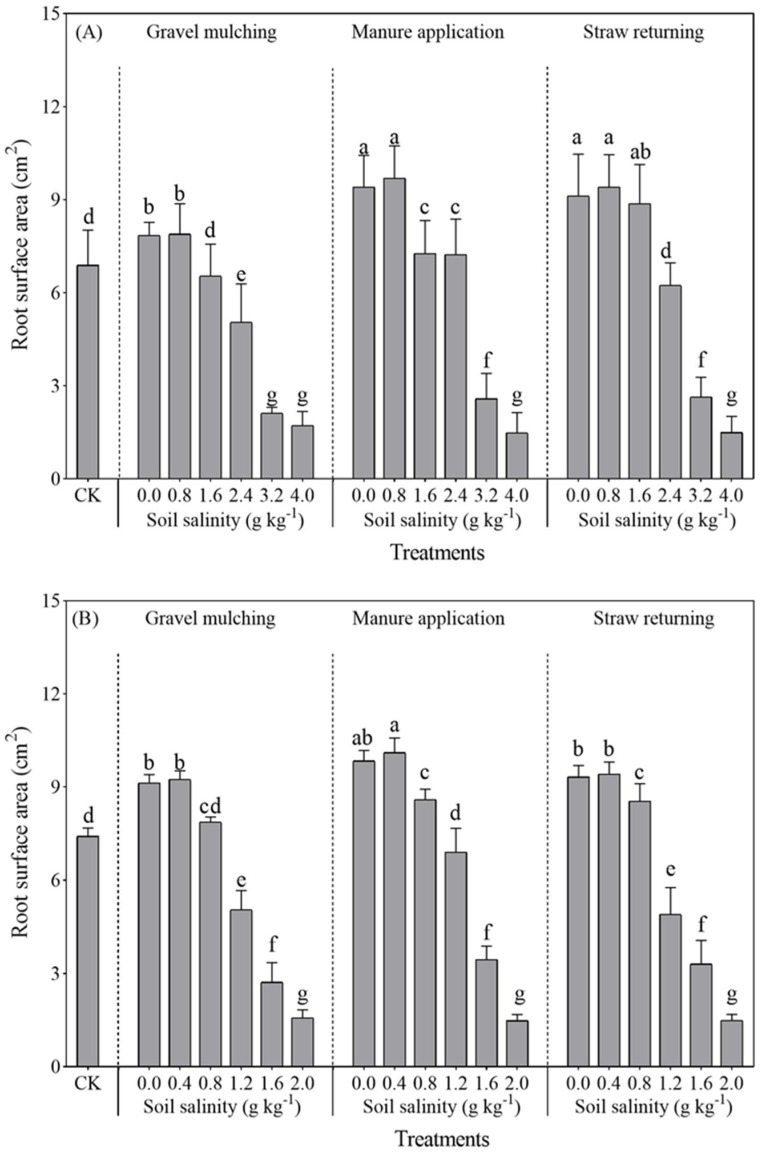
Root surface area of sesbania and hairy vetch under different salinity and saline amelioration measures. (**A**) Sesbania; (**B**) hairy vetch. CK, soil salinity of 0.0 g kg^−1^ without any soil salinity improvement measures. Different lowercase letters indicate that there were significant differences among the 19 treatments at the *p* < 0.05 level.

With the increase of soil salinity, the root surface area of both sesbania and hairy vetch gradually decreased (Figure 8). Compared with CK, the sesbania root surface area at soil salinity of 0.8 g kg^−1^ increased by 11.47%, 14.86%, and 15.60% under gravel mulching, manure application, and straw returning, respectively. The root surface area of hairy vetch was similar to that of sesbania. The root surface area of hairy vetch reached a peak at a soil salinity of 0.4 g kg^−1^. The greatest increase in the root surface area of hairy vetch at a soil salinity of 0.4 g kg^−1^ was observed under manure application, increasing by 10.93% and 11.44% compared with under gravel mulching and straw returning, respectively.

### 2.3. Ion Absorption of Sesbania and Hairy Vetch

#### 2.3.1. Na^+^ Absorption

**Figure 9:**
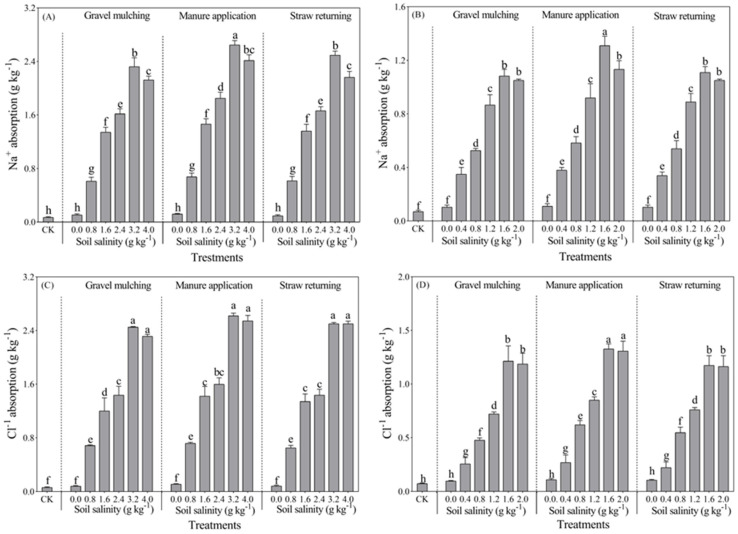
Na^+^ and Cl^−^ absorption of sesbania and hairy vetch under different salinity levels and salinity improvement measures. (**A**,**C**) Sesbania; (**B**,**D**) hairy vetch. CK, soil salinity of 0.0 g kg^−1^ without any soil salinity improvement measures. Different lowercase letters indicate that there were significant differences among the 19 treatments at the *p* < 0.05 level.

Na^+^ absorption of both halophytic crops (sesbania and hairy vetch) reached the maximum value at 3.2 g kg⁻^1^ and 1.6 g kg⁻^1^, respectively (Figure 9). At a soil salinity of 3.2 g kg^−1^, the Na^+^ absorption of sesbania under manure application was 2.71 g kg^−1^, which was 14.7% and 10.95% higher than under gravel mulching and straw returning, respectively. At a soil salinity of 1.6 g kg^−1^, the Na^+^ absorption of hairy vetch under manure application was 1.39 g kg^−1^, which was 24.46% and 22.31% higher than gravel mulching and straw returning. In addition, there was a significant increase in the Na^+^ absorption of both sesbania and hairy vetch under manure application (Figure 9A,B).

#### 2.3.2. Cl^−^ Absorption

With increasing soil salinity, Cl^−^ absorption by sesbania and hairy vetch was the greatest at soil salinities of 3.2 g kg^−1^ and 1.6 g kg^−1^, respectively (Figure 9C,D). The maximum Cl^−^ absorption of hairy vetch and sesbania reached 1.38 and 2.66 g kg^−1^ under VM1.6 and SM3.2, increasing by 85.51% and 92.48% compared with CK, respectively. When soil salinity was 0.0–3.2 g kg^−1^, gravel mulching, manure application, and straw returning treatment gradually increased the Cl^−^ absorption of sesbania, to 2.54, 2.66, and 2.57 g kg^−1^, respectively (Figure 9C). Gravel mulching and manure application slightly decreased the Cl^−^ absorption of sesbania when the soil salinity was more than 3.2 g kg^−1^. The results for hairy vetch were similar to those of sesbania; the absorption of Cl^−^ by hairy vetch gradually increased when soil salinity ranged from 0.0 to 1.6 g kg^−1^. When soil salinity exceeded 1.6 g kg^−1^, all three amendments resulted in a slight decrease in Cl^−^ uptake by hairy vetch (Figure 9D).

#### 2.3.3. K^+^ Absorption

**Figure 10:**
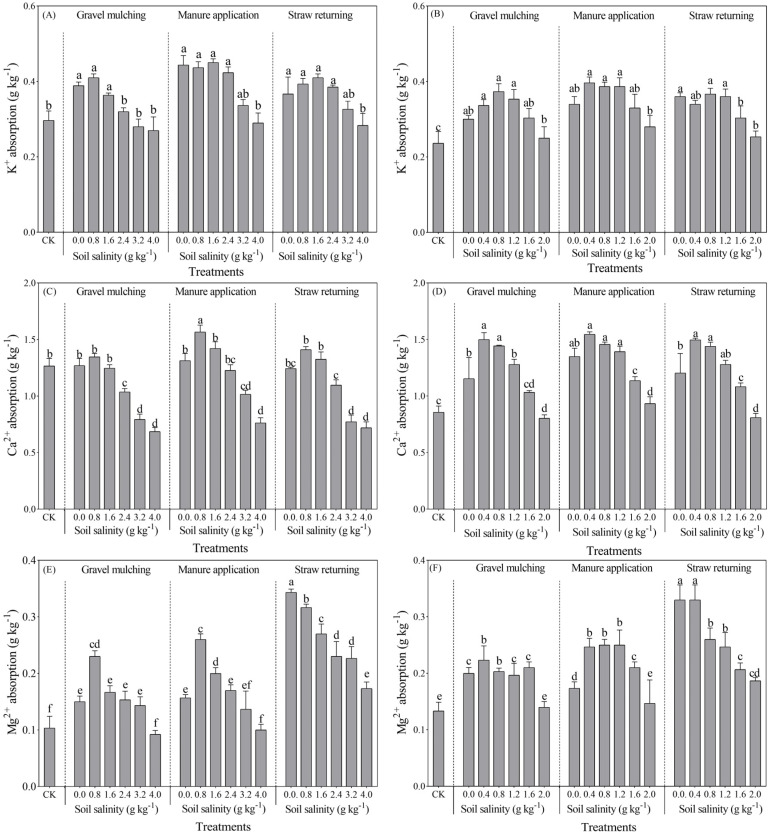
K^+^, Ca^2+^, and Mg^2+^ absorption of sesbania and hairy vetch under different salinity levels and salinity improvement measures. (**A**,**C**,**E**) Sesbania; (**B**,**D**,**F**) hairy vetch. CK, soil salinity of 0.0 g kg^−1^ without any soil salinity improvement measures. Different lowercase letters indicate that there were significant differences among the 19 treatments at the *p* < 0.05 level.

As an essential element for plant growth, the K^+^ absorption of sesbania and hairy vetch varied greatly under gravel mulching. The absorption of K^+^ by sesbania and hairy vetch gradually decreased under gravel mulching when the soil salinity exceeded 0.8 g kg^−1^. However, there was no significant difference in K^+^ absorption between manure application and straw returning treatments for sesbania with a soil salinity less than 1.6 g kg^−1^ (Figure 10A,B). Overall, manure application increased the K^+^ absorption of both sesbania and hairy vetch. At a soil salinity of less than 4 g kg^−1^, the K^+^ absorption of sesbania ranged from 0.26 to 0.45 g kg^−1^. The K^+^ absorption of sesbania reached a maximum value of 0.45 g kg^−1^ under SM0.8. There were no significant differences in the K^+^ absorption of sesbania under gravel mulching, manure application, and straw returning at soil salinities of 3.2 and 4.0 g kg^−1^ (*p* > 0.05). 

#### 2.3.4. Ca^2+^ Absorption

As a plant signal transduction ion, under the manure application, Ca^2+^ absorption by sesbania and hairy vetch was maximized by a soil salinity of 0.8 g kg^−1^ and 0.4 g kg^−1^, respectively (Figure 10C,D). Ca^2+^ absorption was gradually inhibited with the increase of soil salinity. At a soil salinity of 0.8 g kg^−1^, the Ca^2+^ absorption of sesbania reached a peak of 1.62 g kg^−1^ under manure application, which increased by 10.08% and 9.82% compared with gravel mulching and straw returning. At a soil salinity of 4.0 g kg^−1^, the absorption of Ca^2+^ by sesbania was significantly reduced, with the absorption of gravel mulching, manure application, and straw returning being 1.7, 1.98, and 1.78 g kg^−1^; this was 11.76% and 15.25% lower than that of manure application and straw returning, respectively. Similarly, the Ca^2+^ absorption of hairy vetch reached a peak at a soil salinity of 0.4 g kg^−1^. At a soil salinity of 0.4 g kg^−1^, the Ca^2+^ absorption was 1.15, 1.18, and 1.12 g kg^−1^ under gravel mulching, manure application, and straw returning, and there was no significant difference (*p* > 0.05). At a soil salinity of 2.0 g kg^−1^, the Ca^2+^ absorption of hairy vetch under gravel mulching, manure application, and straw returning was 1.38, 1.54, and 1.42 g kg^−1^, representing a reduction of 13.33%, 11.94%, and 12.09% compared to CK.

#### 2.3.5. Mg^2+^ Absorption

Mg^2+^ absorption by sesbania and hairy vetch decreased with increasing soil salinity under straw returning. At a soil salinity greater than 0.8 g kg^−1^, Mg^2+^ absorption by sesbania gradually decreased under gravel mulching and manure application (Figure 10E,F). At a soil salinity of 0.8 g kg^−1^, the Mg^2+^ absorption of sesbania was 0.24, 0.28, and 0.32 g kg^−1^ under gravel mulching, manure application, and straw returning, representing an increase compared with CK, by 16.67%, 22.41%, and 13.46%, respectively. At a soil salinity of less than 1.6 g kg^−1^, the Mg^2+^ absorption of hairy vetch under gravel mulching was lower as compared with manure application and straw returning. At a soil salinity of 2.0 g kg^−1^, the Mg^2+^ absorption of hairy vetch under gravel mulching, manure application, and straw returning was 0.15, 0.16, and 0.19 g kg^−1^, respectively, and there was no significant difference (*p* > 0.05).

## 3. Discussion

Salt stress inhibits plant growth, and with the increase of stress intensity, the cell structure is destroyed, mortality rates increase significantly, and the life cycle cannot be completed [24]. Zhang et al. [25] and Desoky et al. [26] planted oats and wheat, respectively, under 300 mM NaCl stress; oat and wheat seedling growth was significantly inhibited, and plant height and chlorophyll content were significantly reduced. However, the results of the present study showed that the plant height, stem thickness, and leaf area of sesbania and hairy vetch were promoted at low salt concentrations (Figure 1 and Figure 4). Tian et al. [27] obtained the same results by TIR (thermal infrared remote) analysis at different soil salinity levels. Halophytic crops increase the osmotic potential of cells at low salinity levels to prevent water loss and maintain the normal physiological functions of cells [28]. In addition, plants increase plant growth hormone production in response to salinity stress [29], which in turn promotes plant growth, resulting in a corresponding increase in plant height, stem thickness, and leaf area, which is consistent with the results of Ondrasek et al. [30] for the growth characteristics of chrysanthemums under different salinity stresses. In terms of dry matter accumulation, the amount of dry matter of sesbania and hairy vetch gradually decreased with the increase of soil salinity under different amelioration measures. This is due to a series of physiological and biochemical reactions caused by the inhibition of water and nutrients. Under long-term high-salinity stress, the activity of the intracellular synthase of sesbania and hairy vetch was reduced, and osmotic substances were reduced, which finally manifested as the reduction of the growth and biomass accumulation of sesbania and hairy vetch. This finding has also been corroborated in pertinent research concerning the salt tolerance of sunflowers, conducted by Cheng et al. [31]. The results of this study concluded that manure application treatment increased the plant height stem thickness and leaf area of sesbania and hairy vetch, which was attributed to the fact that the addition of organic fertilizers improved the physicochemical properties of the soil so that the plant root system had a better environment, which led to the improvement of the growth and development of the crops [32].

Salt stress affects the plant root system, and root morphology and function reflect crop growth [33]. In this study, the growth and development of the root length, root surface area, and root volume of both sesbania and hairy vetch were inhibited under excessive soil salinity (Figure 5, Figure 6 and Figure 7). This is similar to the conclusions drawn by Silva et al. [34], who grew soybeans at five different salinities. It seems possible that these results, due to salt stress, also led to a reduction in the volume of the root crown and damage to the cellular structure. The root development of sesbania and hairy vetch increased significantly at low salt concentrations compared to CK. A possible explanation for this might be that low concentrations of salt can stimulate plant root growth, and the plant root system adapts to salt stress by altering morphological traits such as root length, root branching, root hair development, and the direction of root growth, changes that optimize the plants’ spatial and temporal structure in saline soils and reduce metabolic costs and the effects of stress [35].

The root system increased significantly under all methods of alleviating soil salinization (gravel mulching, manure application, and straw returning) compared to CK, although manure application was more effective. As soil salinity increased, plant cell membranes were damaged, leading to a decrease in cell membrane permeability [36]. Furthermore, manure application increased soil granular structure and microbial activity, making the soil loose and fertile, which was conducive to root system extension and growth, presenting results similar to those of Ren et al. [37]. Similar results were observed for artemisia when grown in salinity caused by NaCl lower than 0.4 g g^−1^, with greater growth of artemisia annua seeds and lower growth at concentrations above 0.4 g g^−1^ [38]. Likewise, the roots were significantly reduced at concentrations higher than 0.4 g g^−1^ [39].

This study observed that the maximum Na^+^ uptake by sesbania and hairy vetch was 2.71 g kg^−1^ and 1.39 g kg^−1^, respectively, while there was no significant effect on K^+^ absorption in the two species evaluated (Figure 9 and Figure 10). However, Silva et al. [34] found a significant increase in Na^+^ content and a significant decrease in K^+^ content in roots and stems with increasing salt concentration in a soybean salt tolerance test. This was attributed to the similarity between Na^+^ and K^+^ in terms of ionic radius and hydration energy, and the high concentration of Na^+^ in the soil had a significant competitive inhibition on K^+^ uptake [40]. In this study, the growth inhibition of sesbania and hairy vetch was due to the enrichment of Na^+^ in the plants, caused by the increase of soil salinity; a large amount of Na^+^ entered into the plants in the long-term high salt environment, which damaged the membrane structure of the plants and led to the decrease of Na^+^ uptake [34]. The main reason for the increase of Cl^−^ content was that Na+ entered into the plants to reduce the potential of cell membranes, which facilitated the uptake of Cl^−^ in the chemical gradient [40]. The relatively high Cl^−^ accumulation in the root system reflects the active adaptation of saline crops to salt stress. In this study, the capacity of K+ uptake in sesbania was significantly higher than that of hairy vetch. The ion uptake capacity of both sesbania and hairy vetch was significantly higher than that of CK under all methods of soil salinity amelioration (gravel mulching, application of manure, and straw return), but the application of manure was more effective. This indicated that organic manure promoted the growth of saline crops (sesbania and hairy vetch) as well as the uptake of soil ions. This is in agreement with the findings of Song et al. [41] and Liu et al. [42]. This is due to the fact that manure increased the diversity of soil bacterial communities, changed the composition of dominant microbial taxa, improved microbial interactions, and contributed to the maintenance and improvement of soil ecosystem multifunctionality and services [43]. Mg^2+^ and Ca^2+^ are inorganic ions that promote plant growth and do not form a salt effect on plant growth. This study found that Ca^2+^ absorption increased in sesbania and hairy vetch grown under different conditions. This was mainly because both sesbania and hairy vetch are leguminous plants. There might be rhizobacteria with strong nitrogen-fixing ability in their root systems [44]. Organic acids released by root systems also stimulated the release of more Ca^2+^ from soil; thus, the Ca^2+^ absorption of sesbania and hairy vetch increased. Elevated soil salinity might lead to the loss of Ca^2+^ balance in the cytoplasmic membrane. Na^+^ entered plant body and deposited Ca^2+^ on the cytoplasmic membrane. In addition, there was no significant difference in the overall Mg^2+^ absorption of sesbania and hairy vetch. With the increase of soil salinity, Mg^2+^ absorption gradually decreased. A large amount of Na^+^ entered plants, causing damage to the plant membrane structure as well as a loss of membrane selective permeability and a large amount of essential elements from plant cells, resulting in a relative decrease in the amount of Mg^2+^.

Sesbania and hairy vetch possess the capacity to absorb salt ions from the soil, which concurs with the research findings of Tabassum [45]. Panicgrass (*Panicum antidotale*) leads to the maximal accumulation of Na⁺ within the leaves. However, if the residues after harvest are directly returned to the soil, there is a possibility that all the salts will be returned to the soil, thus resulting in ineffective improvement.

## 4. Materials and Methods

### 4.1. Study Site

**Figure 11:**
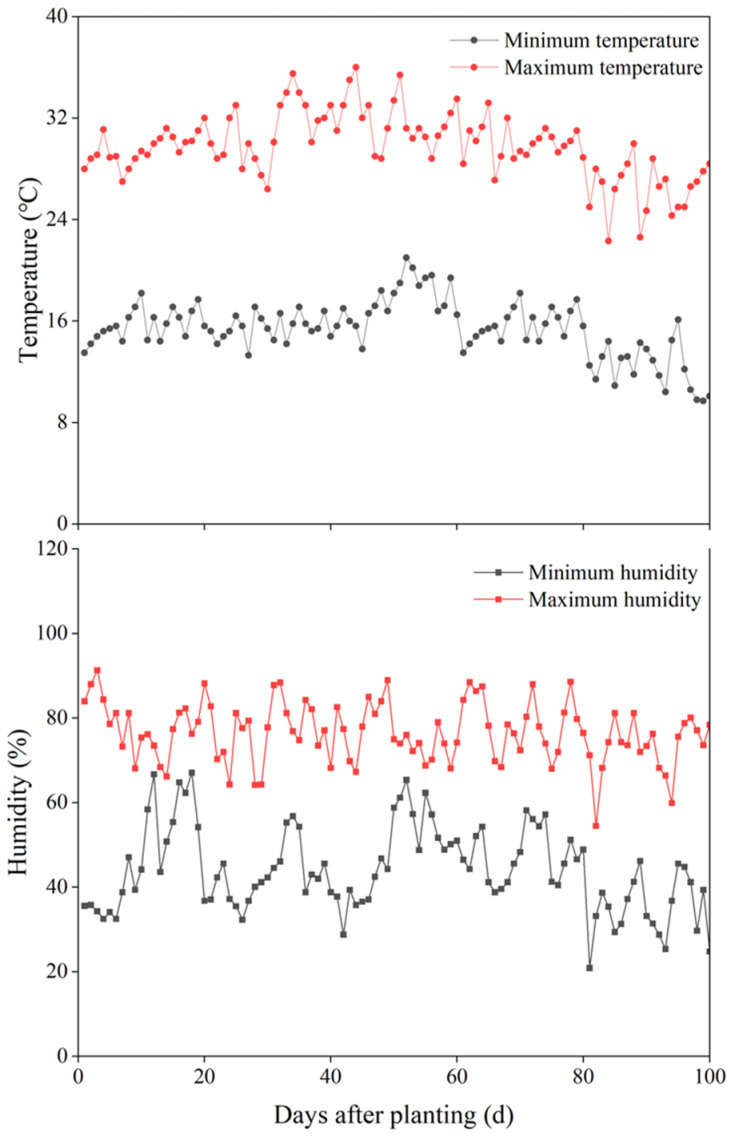
Temperature and humidity in the solar greenhouse.

The experimental soil was collected from the Loess Plateau Ecological Environment Key Field Scientific Observation and Experiment Station in Lanzhou of the Ministry of Agriculture and Rural Affairs. The 0–20 cm soil layer was obtained by using soil augers and shovels in May 2022. Soil surface debris was removed. Large soil particles were crushed. After being dried in a ventilated place and passing through a 2 mm soil sieve, soil was loaded into pots. Soil particle gradation is shown in Table 1 [46]. The soil type was loess, and the texture was loamy soil. The soil water holding capacity was 21.8%. Soil physical and chemical properties are shown in Table 2. The seeds of sesbania and hairy vetch were selected such that they were free from diseases and with uniform size. Greenhouse air temperature and humidity (Figure 11) were monitored daily by a thermometer and humidity meter (DYF60A).

### 4.2. Experimental Design

The pot experiment of sesbania and hairy vetch started in early June 2022. The treatments were a factorial distribution of three factors, including halophytic crops, soil salinization improvement methods, and soil salinity levels. Halophytic crops included sesbania (*Sesbania cannabina*, S) and hairy vetch (*Vicia villosa*, V); soil salinization improvement methods were gravel mulching (C), manure application (M), and straw returning (R). Initial soil salinity was 0.05 g kg^−1^. Sesbania was planted without additional salt application and at additional salt concentrations of 0.8, 1.6, 2.4, 3.2, and 4.0 g kg^−1^. Hairy vetch was planted in the absence of additional salt application and at additional salt concentrations of 0.4, 0.8, 1.2, 1.6, and 2.0 g kg^−1^. No salinity treatment and without any improvement measures was set as the control (CK). A total of 456 pots were used for 38 treatments.

Twenty sesbania or hairy vetch seeds were sown in each pot. The number of seedlings was reduced to 15 seedlings in each pot after germination, in order to avoid competition between plants for soil nutrients. The specific experimental treatments are shown in Table 3. After that, NaCl was added to the soil in two additions with irrigation water. Among them, 6.9, 30.8, 54.7, 78.7, and 102.6 mM NaCl was added each time to the soil in which hairy vetch was planted. In addition, 30.8, 78.8, 126.5, 174.4, and 222 mM NaCl was added each time to the soil in which sesbania was planted. The seeds of sesbania and hairy vetch were evenly sown in pots covered with 2 cm of soil. The soil bulk density was 1.35 g cm^−3^, the diameter of the pots was 22.5 cm, and the height of pots was 21.5 cm. The soil depth and weight of each pot were 16 cm and 7 kg, respectively. Gravel was collected from the Yellow River beach in China, sieved through an 8 mm sieve. The quantity of gravel was determined according to 20% (1.6 kg) of the volume of soil within the pots. The gravel was used as mulch for the surface soil layer. The pot treated with manure received 270 g of sheep manure with a pH of 7.6, N of 1.8%, P_2_O_5_ content of 1.4%, and K_2_O of 0.9%. In the straw returning treatment, 150 g of 2–3 cm wheat straw in length was added to each pot. The depth of wheat straw returning over in the experimental pot was 10 cm [47]. The pots were placed in order of soil salinity from highest to lowest according to the same amelioration measure. The pots had little impact on each other, as they were placed 10 cm apart. Sesbania and hairy vetch at prime bloom stage were harvested on the 120th day of sowing (in early October).

In the early stage, to ensure the emergence rate, each pot was irrigated with 1200 mL of water. After the emergence was completed, the irrigation volume took a soil water content of 90% of the field capacity as the control line. Irrigation was carried out to the control line every 2 d by the weighing method. No bacterial inoculum was used in this experiment.

### 4.3. Measurement Indicators

Plant height and stem diameter were measured at seedling, branching, current bud, and prime bloom stages of sesbania and hairy vetch, with five replicates per treatment. Plant height was determined using a tape measure from the base of the stem to the top of the plant. Stem diameter was measured in both longitudinal and transverse directions using a vernier caliper at 2 cm from the base of the stem.

Plant samples of sesbania and hairy vetch were taken at the prime bloom stage. The organs (leaves, stems, and roots) of plant samples were separated, packed into paper bags, and then dried to constant weight in a constant temperature oven at 80 °C. The dry matter mass of samples was measured using an electronic scale with an accuracy of 0.01 g.

The leaf area was determined using a leaf area meter (YMJ-A, Hangzhou, China) at the branching, current bud, and prime bloom stages for both sesbania and hairy vetch.

The intact roots of sesbania and hairy vetch were obtained and cleaned. Root length, root volume, root surface area, and root diameter were analyzed using root analysis software (WinRHIZO, Quebec, QC, Canada).

Soil and plant K^+^ and Na^+^ contents were determined by the flame photometer method. Soil and plant Ca^2+^ and Mg^2+^ were determined by an atomic absorption spectrophotometer [48,49]. Soil and plant Cl^−^ was measured by AgNO_3_ titration.

### 4.4. Statistical Analysis

Excel 2019 and Origin 2018 were used for data processing. SPSS 26.0 software was used for significant difference analysis. Regression analysis was used for multiple comparisons between treatments. Origin 2018 was used for plotting.

## 5. Conclusions

Compared with treatments without improvement measures, gravel mulching, manure application, and straw returning significantly promoted plant height, stem diameter, leaf area, root–shoot ratio, and root growth of sesbania and hairy vetch at soil salinities lower than 2.4 g kg^−1^ and 1.2 g kg^−1^. The absorption amounts of sesbania and hairy vetch for Na⁺ and Cl^−^ were the greatest at a soil salinity of 3.2 g kg^−1^ and 1.6 g kg^−1^, respectively. Subsequently, the increase in soil salinity led to a decrease in the absorption amounts of Na⁺ and Cl^−^ by sesbania and hairy vetch. The Ca^2+^ absorption of sesbania and hairy vetch was gradually suppressed. The maximum absorption of Na^+^ and Cl^−^ by sesbania and hairy vetch under manure application was favorable for the improvement of soil salinity. Manure application was superior to the gravel mulching and straw returning; manure application at the same soil salinity resulted in a maximum increase in the absorption of salt ions by sesbania and hairy vetch by 10.4% and 6.7% compared with gravel mulching and straw returning. Therefore, application of manure with sesbania and hairy vetch is more beneficial in reducing soil salinity than gravel mulching and straw return. Sesbania and hairy vetch were more beneficial in ameliorating soil salinity at levels of 3.2 g kg^−1^ and 1.6 g kg^−1^, respectively. Although this study concluded that manure application exerted a better effect on soil salinization alleviation, further research is still urgently required to determine whether manure application could remain efficient under lower or higher soil salinity levels with other halophytic crops.

## Figures and Tables

**Table 1:** Grain size gradation of test soil, gravel, and sand.

Particle Size (mm)	<2.00	<1.00	<0.05	<0.01	<0.001
Soil (%)	100	98.37	82.41	21.33	9.06

**Table 2:** Physical and chemical properties of soil for testing in different soil layers.

Soil Depth (cm)	Organic Matter (g kg^−1^)	Na^+^ (g kg^−1^)	K^+^ (g kg^−1^)	Ca^2+^ (g kg^−1^)	Mg^2+^ (g kg^−1^)	Cl^−1^ (g kg^−1^)	pH	EC (mS cm^−1^)
0–20	9.40	0.13	0.27	0.38	0.13	0.11	7.79	0.66

**Table 3:** Experimental treatments and abbreviations for each treatment.

Crop	Salt Addition (g kg^−1^)	Improvement Measures	Treatments	Crop	Salt Addition (g kg^−1^)	Improvement Measures	Treatments
Hairy vetch (V)	0	None	CK	Sesbania (S)	0	None	CK
		Gravel mulching (C)	VC0			Gravel mulching (C)	SC0
		Manure application (M)	VM0			Manure application (M)	SM0
		Straw returning (R)	VR0			Straw returning (R)	SR0
	0.4	Gravel mulching (C)	VC0.4		0.8	Gravel mulching (C)	SC0.8
		Manure application (M)	VM0.4			Manure application (M)	SM0.8
		Straw returning (R)	VR0.4			Straw returning (R)	SR0.8
	0.8	Gravel mulching (C)	VC0.8		1.6	Gravel mulching (C)	SC1.6
		Manure application (M)	VM0.8			Manure application (M)	SM1.6
		Straw returning (R)	VR0.8			Straw returning (R)	SR1.6
	1.2	Gravel mulching (C)	VC1.2		2.4	Gravel mulching (C)	SC2.4
		Manure application (M)	VM1.2			Manure application (M)	SM2.4
		Straw returning (R)	VR1.2			Straw returning (R)	SR2.4
	1.6	Gravel mulching (C)	VC1.6		3.2	Gravel mulching (C)	SC3.2
		Manure application (M)	VM1.6			Manure application (M)	SM3.2
		Straw returning (R)	VR1.6			Straw returning (R)	SR3.2
	2	Gravel mulching (C)	VC2.0		4	Gravel mulching (C)	SC4.0
		Manure application (M)	VM2.0			Manure application (M)	SM4.0
		Straw returning (R)	VR2.0			Straw returning (R)	SR4.0

## Data Availability

The original contributions presented in this study are included in this article; further inquiries can be directed to the corresponding authors.
